# Longevity extension in Drosophila through gut-brain communication

**DOI:** 10.1038/s41598-018-25382-z

**Published:** 2018-05-30

**Authors:** Susan Westfall, Nikita Lomis, Satya Prakash

**Affiliations:** Biomedical and Cell Therapy Research Laboratory, Department of Biomedical Engineering, McGill University, 3775 University Street, Room 322, Montreal, QC H3A2B4 Canada

## Abstract

Aging and chronic disease development are multifactorial processes involving the cumulative effects of metabolic distress, inflammation, oxidative stress and mitochondrial dynamics. Recently, variations in the gut microbiota have been associated with age-related phenotypes and probiotics have shown promise in managing chronic disease progression. In this study, novel probiotic and synbiotic formulations are shown to combinatorially extend longevity in male *Drosophila melanogaster* through mechanisms of gut-brain-axis communication with implications in chronic disease management. Both the probiotic and synbiotic formulations rescued markers of metabolic stress by managing insulin resistance and energy regulatory pathways. Both formulations also ameliorated elevations in inflammation, oxidative stress and the loss of mitochondrial complex integrity. In almost all the measured pathways, the synbiotic formulation has a more robust impact than its individual components insinuating its combinatorial effect. The concomitant action of the gut microbiota on each of the key risk factors of aging and makes it a powerful therapeutic tool against neurodegeneration, diabetes, obesity, cardiovascular disease and other age-related chronic diseases.

## Introduction

The gut microbiota is complex ecosystem of bacteria, fungi and microorganisms residing in the gastrointestinal tract (GIT), which impart many health benefits onto the host including digestion of otherwise indigestible fibers, synthesis of vitamins and minerals, gastrointestinal motility, anti-inflammatory, antioxidant and energy regulating properties^1^. Distinct variations in the composition of the gut microbiota in the elderly have been identified and could contribute to frailty, disease development and aging itself^2,3^. Aging is a multifactorial process that remains to be fully understood though encompasses several physiological changes due to increased vulnerability to environmental stresses^4^. Previously, lifespan extension was believed to be coupled with chronic disease; however, it has recently been discovered that organisms with mutations that prolong life also postpone age-related diseases^5^ indicating that the mechanisms of aging and chronic disease development are intertwined and that therapies that promote longevity could be used to prevent chronic diseases and vice versa.

There are many theories of aging though no single theory can fully explain the aging process. Aging is likely due to an intersection of hereditary, epigenetic and stochastic environmental events^6^ causing accumulation of damage over time. Metabolic pathways^7^, neuroendocrine mediators^8^, immunosenescence^9^ and the accumulation of oxidants^10^ all contribute to various aging hypotheses. The most robust and experimentally verified method to increase longevity in model organisms is calorie restriction^11^ indicating the importance of nutrient assimilation in the gastrointestinal tract (GIT) in longevity.

The composition of gut microbiota is linked to healthy aging^12–14^ and a diet rich in probiotics and prebiotics may help prevent chronic age-related disease^15,16^. Changes in the gut microbiota of aging individuals has a high inter-individual variability due to disease manifestation, medication, diet and environmental exposure^13^. In general, aging subjects have a decline in the phyla Firmicutes, elevation in Bacteriodetes, reduction of *Bifidobacteria* spp., elevation in the proinflammatory *Proteobacteria* accompanied by a decline in overall diversity, which is associated with various health risks and fraility^13,14,17–19^. Indeed, a general decrease in the level of short-chain fatty acids (SCFAs) is apparent in aging individuals^2^ which is linked to inflammation and adipose tissue dysregulation^20^.

The gut-brain-axis (GBA) is a bidirectional communication system between the GIT microbiota and the brain including various metabolic, immunological, endocrine and neuronal signals derived from individual bacterial cells and their metabolites^21^. Through this axis, the gut microbiota was recently identified as a target for therapeutic intervention against age-related diseases. For example, several probiotic bacteria have shown beneficial effects in managing symptoms of neurodegeneration^22^. *Lactobacillus brevis* was shown to reduce age-related colitis, memory impairments and NF-κB activation in aging mice^23^. In another aging mouse model, *L. fermentum* MTCC 5898 in fermented milk elevated immunity, antioxidant defense and reduced *E. coli* populations^24^. Similarly, *B. lactis* reduced the level of low-grade inflammation and promoted longevity in mice^25^. Probiotic supplementation was also shown to induce longevity in *C. elegans* by stimulating the innate immune response and reducing oxidative stress^26^. In another study, *L. salivarius* isolated from centarians’ fecal samples extended the lifespan of *C. elegans* by 11.9% in a calorie-restriction dependent manner^27^. Clearly, the prolongevity mechanisms of lower organisms’ parallel mammals making *Drosophila melanogaster* a good model for understanding the impact of probiotic therapy on longevity and the mechanisms of aging.

The present study describes how a novel probiotic and synbiotic formulation impacts *Drosophila melanogaster* longevity through mechanisms of the GBA. It was previously shown that the probiotic and synbiotic formulation used in the present study has beneficial effects on aging^28^. The present probiotic and synbiotic formulations showed combinatorial action on reducing markers of physiological stress, oxidative stress, inflammation and mitochondrial ETC complex integrity therefore targeting most of the main aging mechanisms. This action benefits not only longevity but would prevent many age-related chronic diseases that are associated with the aforementioned states.

## Results

### The probiotic and synbiotic formulation has beneficial effects on physiological metabolic markers in aging *Drosophila melanogaster*

**Figure 1:**
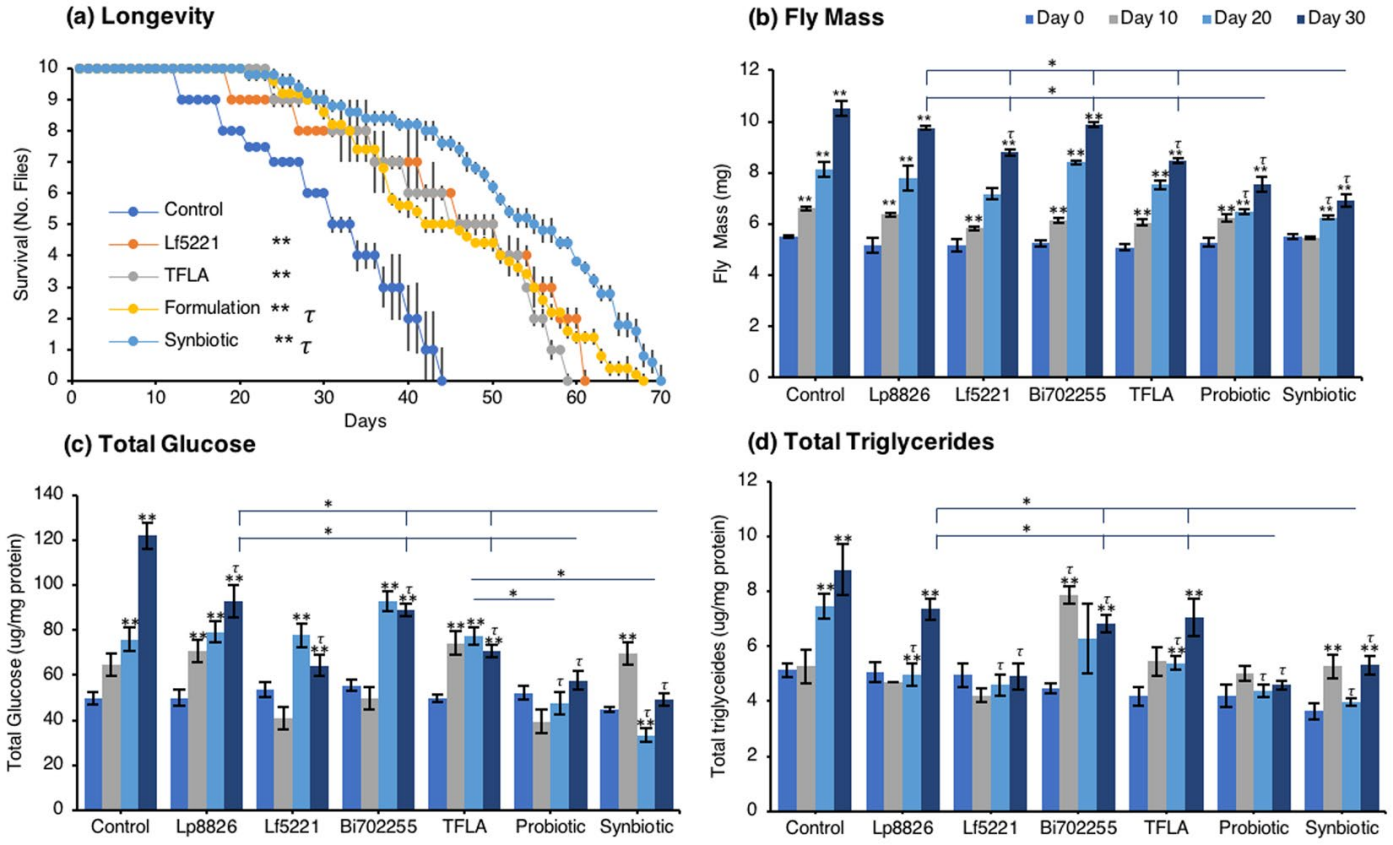
Supplementation with probiotics, a probiotic formulation, the prebiotic or their combination (synbiotic) impacts longevity and metabolic parameters in aging *Drosophila melanogaster*. Longevity (**a**) was assessed in *Drosophila* by counting daily living flies over time. Metabolic parameters including (**b**) weight, (**c**) total glucose and (**d**) total triglycerides were measured in aging flies at days 0, 10, 20 and 30. Each group contained n = 5 independent groups and significance is indicated as stars (*) relative to the control day 0 group with *p < 0.05 and **p < 0.01 and as tau (*τ*) as relative to the no-treatment control at the same time point where ^*τ*^p < 0.05.

Longevity in male Oregon-R *Drosophila* was assessed by counting the number of living flies daily over 30 days (Fig. 1a). In *Drosophila* on a normal diet, the maximal lifespan was at 40 days. In flies treated with the Lf5221 alone or TFLA alone, there was a significant 16- and 14-day increase in longevity while flies treated with the probiotic or synbiotic formulations had a 24- and 26- day increase in longevity. The physiological metabolic parameters of *Drosophila* were determined every ten days for thirty days in aging *Drosophila* exposed to either probiotic, prebiotic, the probiotic or synbiotic formulation beginning at the first day after eclosion. As expected, the total weight of *Drosophila* in the untreated controls showed a time-dependent elevation (Fig. 1b). This trend was observed in all the groups; however, by day 30, the probiotic and synbiotic formulation invoked a significant reduction in total weight compared to controls, the individual probiotics and TFLA-treated group (Fig. 1b). Similarly for the total glucose measurement in aging *Drosophila*, there was a time-dependent increase in total glucose in control *Drosophila* peaking with a 2.5-fold increase by day 30 (Fig. 1c). Each of the individual probiotics and TFLA prebiotic showed similar time-dependent increase in total glucose levels however at day 30, there was a reduction in total glucose in all groups. Notably, both the probiotic and synbiotic formulation completely rescued the elevation in total glucose by day 30, which was significantly lower than Lp8826, Bi702255 and the TFLA-treated groups (p < 0.01). Finally, variation in total triglyceride levels (F (3,18) = 5.34) were elevated by 1.7-fold in untreated controls by day 30 (Fig. 1d, p < 0.01). By day 30, there were reductions in total triglyceride levels in the Lf5221, Bi702255, probiotic and synbiotic formulation groups, with Lf5221 and the probiotic formulation completely reducing the total triglyceride levels to the level of day 0 flies. Overall, the probiotic formulation was the most effective at reducing all the physiological markers of metabolic stress in *Drosophila*, while the synbiotic formulation showed almost as effective results.

### Insulin-like signaling in aging *Drosophila melanogaster* is positively affected by probiotic and/or prebiotic treatment

**Figure 2:**
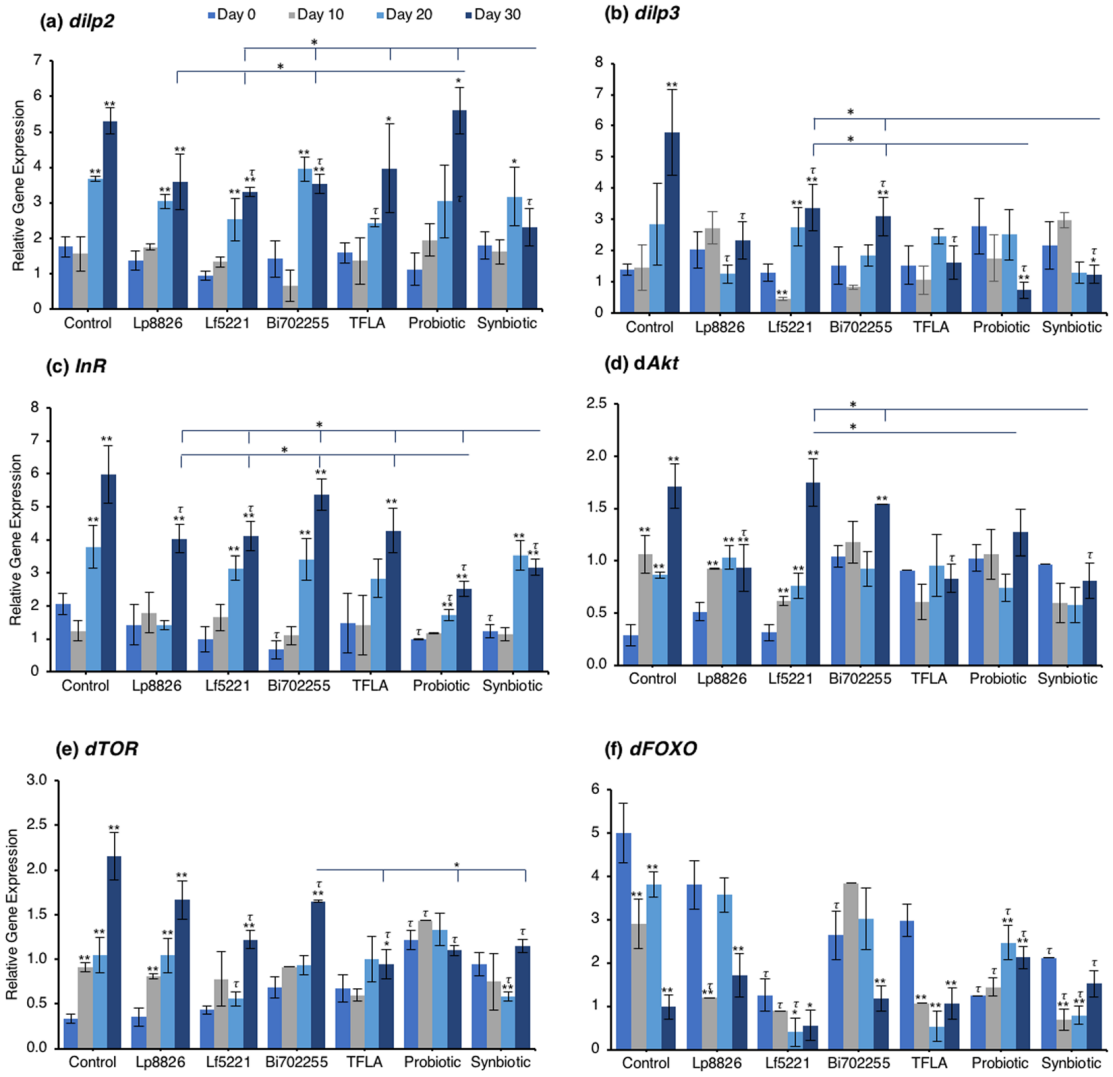
Assessment of genetic markers of diabetes in aging *Drosophila melanogaster* supplemented with the probiotic and synbiotic formulation. At 0, 10, 20 and 30 days, the expression of several insulin-signaling genes, namely (**a**) *Drosophila insulin-like peptide* (*dilp*)2, (**b**) *dilp*3, (**c**) insulin receptor (InR), (**d**) dAkt, (**e**) dTOR and (**f**) dFOXO. Each group contained n = 5 independent groups and significance is indicated as stars (*) relative to the control day 0 group with *p < 0.05 and **p < 0.01 and as tau (*τ*) as relative to the no-treatment control at the same time point where ^*τ*^p < 0.05.

To assess the underlying genetic regulatory mechanisms of metabolism affected by the prebiotic or probiotic supplementation, the expression of a battery of insulin signaling genes was assessed using real-time PCR. *Drosophila insulin-like peptide* (*dilp*)2 expression was elevated at days 20 and 30 in control flies (Fig. 2a; p < 0.01). At day 20, only TFLA supplementation lowered *dilp*2 expression while at day 30, Bi702255 and synbiotic formulation reduced *dilp2* expression by 33% and 57%, respectively (p < 0.01). Notably, only synbiotic formulation was able to rescue *dilp*2 expression, which was significantly lower than any of the other treatment groups. Similarly, *dilp*3 expression was elevated at day 30 in the control group (Fig. 2b; p < 0.01), which was significantly reduced by all groups. Supplementation with Lp8826 and TFLA rescued *dilp3* expression to the level of day 0 controls while the probiotic and synbiotic formulation reduced *dilp*3 expression further; a decrease that was significantly lower than Lf5221 and Bi702255 at day 30. The insulin receptor (InR) was likewise elevated at days 20 and 30 in control *Drosophila* (Fig. 2c; p < 0.01). At day 20, only the probiotic formulation significantly reduced InR expression while at day 30, Lp8826, Lf5221, the probiotic and synbiotic formulation elicited significant reductions (p < 0.01). Notably, the probiotic formulation reduced InR level at day 30 to a greater extent than all the other groups while the synbiotic formulation was lower than all groups spare the probiotic formulation.

Examining the insulin signaling pathways downstream of the insulin receptor, similar yet intriguing results were obtained. dAkt expression was elevated in the aging control *Drosophila* from day 10 to day 30 (Fig. 2d; p < 0.01). At day 30, supplementation with Lp8826, TFLA and the synbiotic formulation significantly reduced dAkt expression (p < 0.05). Notably, TFLA, the probiotic and synbiotic formulations completely rescued dAkt levels at day 30 with the synbiotic formulation reducing dAkt expression greater than Lf5221 and Bi702255. Directly downstream of dAkt is dTOR, whose expression was likewise elevated from day 10 to 30 in control *Drosophila* (Fig. 2e; p < 0.01). Only Lp8826 did not reduce dTOR levels at day 30 while TFLA, the probiotic and synbiotic formulation completely rescued expression. Finally, dFOXO, a transcription factor downregulated by dAkt, was downregulated in control aging *Drosophila* from day 10 to day 30 (Fig. 2f; p < 0.01). Only the probiotic and synbiotic formulations were able to elevate dFOXO expression levels and only the synbiotic formulation completely rescued its expression to the level of day 0 controls. Overall, the individual probiotics and TFLA had differential yet positive impacts on the insulin-signaling pathways in aging *Drosophila*; however, the probiotic and synbiotic formulations demonstrated combinatorial effects on rescuing the underlying insulin signaling dysregulation in aging *Drosophila*.

### Fatty acid metabolism is benefited from supplementation of the probiotic and synbiotic formulation in aging *Drosophila melanogaster*

**Figure 3:**
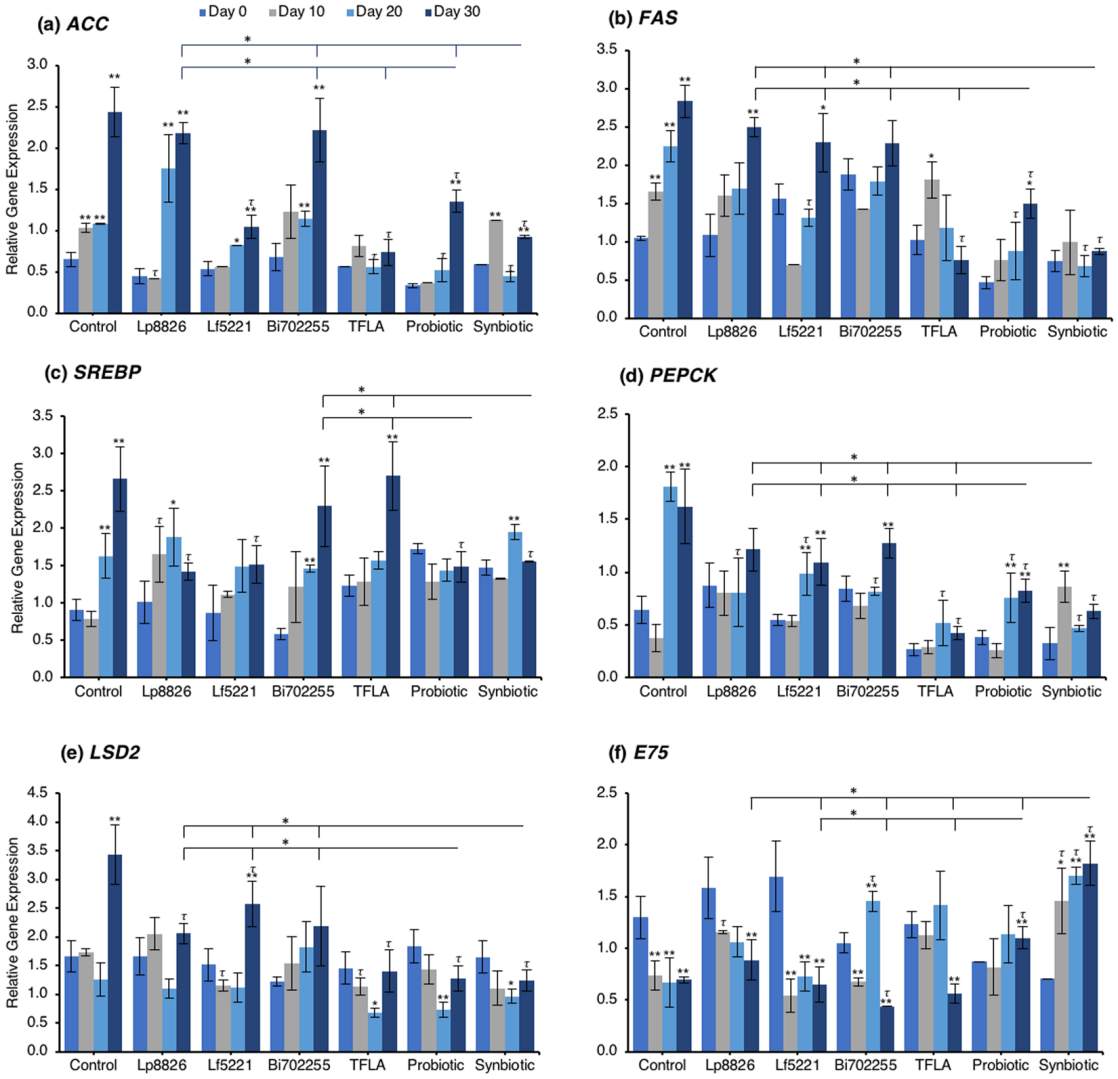
Genetic markers of obesity in aging *Drosophila melanogaster* supplemented with the probiotic or synbiotic formulation. At 0, 10, 20 and 30 days, expression of fat metabolism genes, namely (**a**) acetyl-CoA carboxylase (ACC), (**b**) fatty acid synthase (FAS), (**c**) sterol-regulatory element binding protein (SREBP), (**d**) phospholenol pyruvate carboxykinase (PEPCK) (**e**) lipid storgae droplet (Lsd) 2 and (f) E75. Each group contained n = 5 independent groups and significance is indicated as stars (*) relative to the control day 0 group with *p < 0.05 and **p < 0.01 and as tau (*τ*) as relative to the no-treatment control at the same time point where ^*τ*^p < 0.05.

To determine the mechanism through which the total triglycerides were elevated in aging *Drosophila* and how the probiotics alleviated this effect, the expression of several lipogenesis and adipogenesis regulatory factors was assessed. Acetyl-CoA carboxylase (ACC) and fatty acid synthase (FAS) are both lipogenic factors activated by the transcription factor sterol regulatory element binding protein (SREBP). Expression of both ACC (Fig. 3a) and FAS (Fig. 3b) were significantly elevated from day 10 to day 30 (p < 0.01) indicating an elevation in lipogenesis in aging control *Drosophila*. At day 30, *ACC* expression was reduced by Lf5221, TFLA, the probiotic and synbiotic formulation with the synbiotic formulation having a greater effect than the probiotic formulation (p < 0.05). *FAS* expression was only reduced by TFLA, the probiotic and synbiotic formulations at day 30 where TFLA and the synbiotic formulations rescued *FAS* expression to the level of day 0 controls. *SREBP* expression followed the same trend (Fig. 3c). The elevation at days 20 and 30 (p < 0.01) was reduced by Lp8826 and Lf5221 and rescued by the probiotic and synbiotic formulations at day 30 (p < 0.01). Phosphoenolpyruvate carboxykinase (PEPCK) is a major regulator of gluconeogenesis and like the lipogenesis genes, increased in expression at days 20 and 30 in control *Drosophila* (Fig. 3d; p < 0.01). At day 30, *PEPCK* expression was reduced by TFLA, the probiotic and synbiotic formulations with TFLA and the synbiotic formulation rescuing *PEPCK* expression to the level of day 0 controls (p < 0.01). Lipid storage droplet (LSD) 2 is expressed in the fat body and controls triglyceride accumulation. *LSD2* expression was elevated at day 30 in control *Drosophila* (Fig. 3e; p < 0.01) and rescued by Lp8826, TFLA, the probiotic and synbiotic formulations (p < 0.01). Finally, E75 a transcriptional target of the *Drosophila* peroxisome proliferator-activated receptor (PPAR)γ homolog responsible for adipogenesis and insulin sensitivity. *E75* expression was significantly reduced at days 10 through 30 in aging controls (p < 0.01), an effect that was reduced by Bi702255 at day 30 and elevated only by the probiotic and synbiotic formulation at day 30 with the synbiotic formulation having a greater impact than the probiotic formulation (p < 0.05).

### Inflammatory markers elevated with age are reduced by combinatorial prebiotic and probiotic treatment

**Figure 4:**
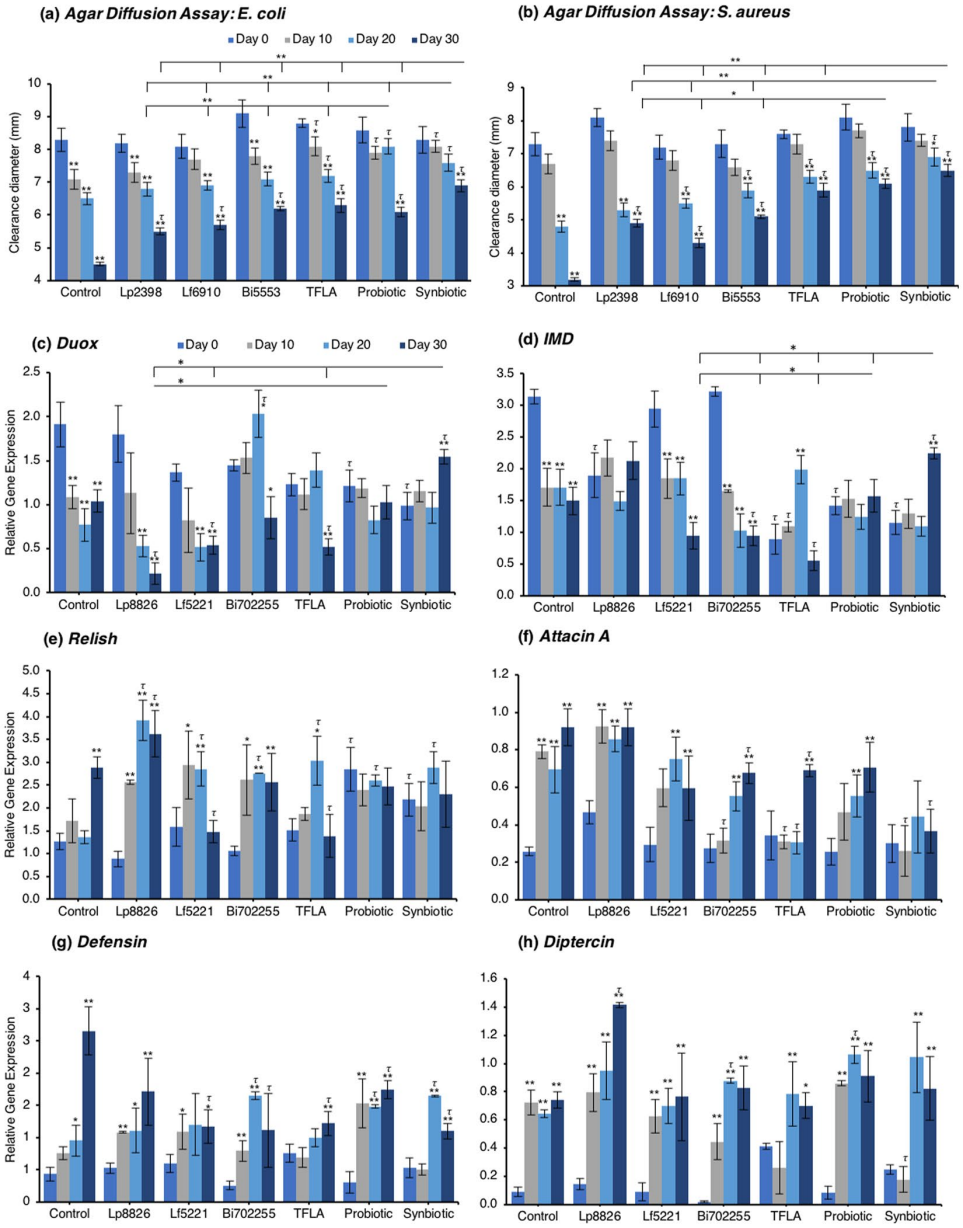
Age-related variations in innate immunity factors in *Drosophila melanogaster* are ameliorated by the synbiotic formulation. At days 0, 10, 20 and 30, *Drosophila melanogaster* on media supplemented with the individual probiotics, prebiotic or a combination of the probiotics and/or prebiotics were tested for their immunological response. The agar diffusion test measuring the production of antimicrobial factors was performed on plates with lawns of (**a**) *E. coli* and (**b**) *S. aureus* pathogenic species. Gene expression of several immunological genes were also assessed including (**c**) dual oxidase (duox), (**d**) immune deficiency (IMD), (**e**) Relish, (**f**) Attacin A, (**g**) Defensin and (**h**) Diptercin. Each group contained n = 5 independent groups and significance is indicated as stars (*) relative to the control day 0 group with *p < 0.05 and **p < 0.01 and as tau (*τ*) as relative to the no-treatment control at the same time point where ^*τ*^p < 0.05.

The agar diffusion test measures the amount of secretable immunogenic factors and their immunogenicity against a pathogenic lawn on a nutrient agar plate. Against *E. coli*, control *Drosophila* experienced a large reduction in immunogenicity as the clearance diameter was reduced by 47% from day 0 to day 30 (Fig. 4a; p < 0.01). At day 20, TFLA, the probiotic and synbiotic formulations improved the clearance diameter (p < 0.01) while at day 30, all the supplementation groups had a positive effect. At day 20 and 30, the synbiotic formulation was the most effective at improving the clearance diameter supporting its combinatorial effect. A similar trend was noted for the clearance diameter on a *S. aureus* plates where control *Drosophila* demonstrated a 56% decrease in clearance diameter from day 0 to day 30 (Fig. 4b; p < 0.01). All supplementation groups elicited a positive effect at days 20 and 30; however, both the probiotic and synbiotic formulations had a greater effect than the individual probiotics or TFLA alone.

Concerning regulation of the immunological genetic markers, expression of *dual oxidase* (*Duox*), the primary innate immune response that stimulates ROS release following a pathogenic insult, was reduced at days 10 through 30 in the untreated *Drosophila* (Fig. 4c; p < 0.01). At day 30, *Duox* expression was rescued by the probiotic and synbiotic treatment compared to the day 0 control, but only the synbiotic formulation at day 30 improved *Duox* expression compared to the untreated control. *Immune deficiency* (*IMD*) is another innate immune responder though is slightly more sensitive to gram negative bacteria. *IMD* expression was reduced in control *Drosophila* at days 10 through 30 (Fig. 4d; p < 0.01) and at day 30, was only elevated by the synbiotic formulation. Nevertheless, due to variations in baseline expression, Lp8826, TFLA the probiotic and synbiotic formulation all effectively rescued *IMD* expression at day 30 (p > 0.05). *Relish* is a cytokine-like factor downstream of IMD pathway whose expression was upregulated at day 30 in control *Drosophila* (Fig. 4e; p < 0.01). The impact of the probiotic bacteria was limited and varied though both the probiotic and synbiotic formulations eliminated any variation in Relish expression in the aging *Drosophila*.

The antimicrobial peptides (AMPs) are the effector molecules of the innate immune system induced downstream of the *IMD* and Toll-pathways. *Attacin A* transcription is activated both by gram-positive and gram-negative bacterial insults and in the present model, increased dramatically in aging control *Drosophila* in days 10 through 30. (Fig. 4f; p < 0.01). At day 30, *Attacin A* expression was reduced by Bi702255, TFLA and the synbiotic formulation with the latter rescuing expression to the level of day 0 controls (p < 0.01). *Defensin* is activated downstream of the toll-pathway and gram-positive bacterial insults. Like *Attacin A*, *Defensin* expression was elevated in aging control *Drosophila* (Fig. 4g; p < 0.01) and reduced at day 30 by all supplementation groups except Lp8826. Finally *Diptercin*, activated downstream of IMD from a gram-negative challenge, was also elevated in aging control *Drosophila* on days 10 through 30 (Fig. 4h; p < 0.01), though there was little impact of any probiotic and/or prebiotic treatment.

### Age-related elevations in oxidative stress are alleviated by probiotic and prebiotic treatment

**Figure 5:**
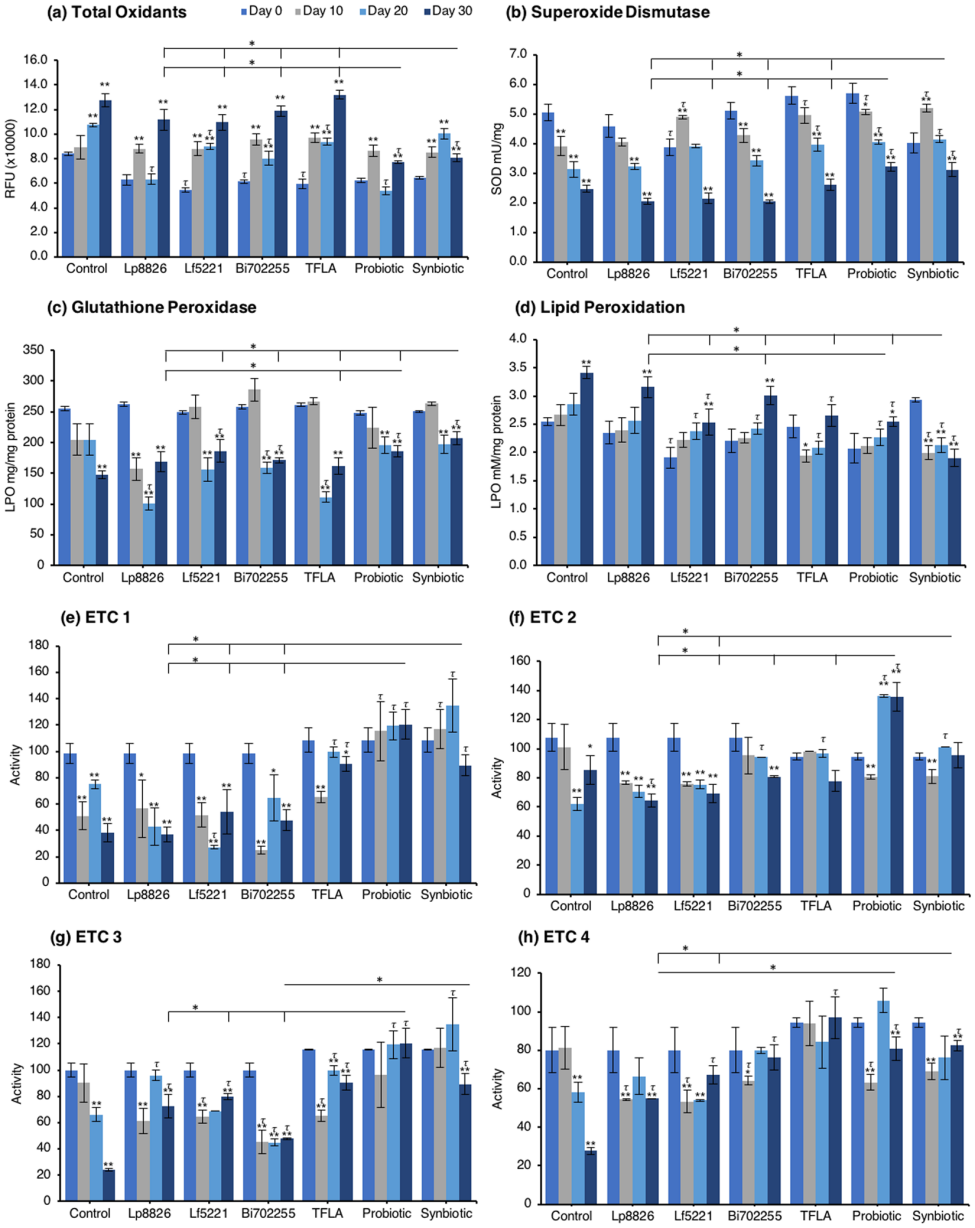
The probiotic and synbiotic formulations have combinatorial effects on markers of oxidative stress. *Drosophila melanogaster* were supplemented with the individual probiotic, prebiotic, the probiotic or synbiotic formulation and the levels of (**a**) total oxidants, (**b**) superoxide dismutase activity, (**c**) glutathione peroxidase activity and (**d**) lipid peroxidation were measured at 0, 10, 20 and 30 days. The activity of ETC (**e**) complex 1 (NADH coenzyme Q reductase), (**f**) complex 2 (succinate dehydrogenase), (**g**) complex 3 (cytochrome bc1 complex) and (**h**) complex 4 (cytochrome c oxidase) was also assessed using various biochemical assays. Each group contained n = 5 independent groups of 25 flies and significance is indicated as stars (*) relative to the control day 0 group with *p < 0.05 and **p < 0.01 and as tau (*τ*) as relative to the no-treatment control at the same concentration where ^*τ*^p < 0.05.

The gradual accumulation of oxidative stress is a major contributor to aging and in the present model an increase in oxidants and corresponding decrease in antioxidant enzyme activity was dually observed. The level of total oxidants was significantly elevated in control *Drosophila* at days 20 and 30 peaking at a 50% increase by day 30 (Fig. 5a; p < 0.01). Supplementation only with the probiotic or synbiotic formulation reduced the total oxidant load at day 30, which for both groups was significantly greater than the effect of the individual probiotics or TFLA (p < 0.05). Notably, Lf5221, Bi702255 and TFLA all elicited beneficial reductions in total oxidants at day 20. Superoxide dismutase (SOD) is essential for converting superoxide anions in the less harmful hydrogen peroxide. SOD activity was reduced by 50% in control *Drosophila* from day 0 to day 30 (Fig. 5b; p < 0.01), an effect that was only positively impacted by the probiotic or synbiotic formulations at days 10, 20 and 30 (p < 0.05). The TFLA formulation also improved SOD activity at days 10 and 20; however, to a lesser extent than both the probiotic and synbiotic formulations. Similarly, glutathione peroxidase (GPx) activity which converts hydrogen peroxide to water, was reduced by 42% in aging control *Drosophila* from day 0 to day 30 (Fig. 5c; p < 0.01). At day 30, Lf5221, the probiotic or synbiotic formulation had beneficial effects, with the synbiotic formulation having more significant impact than any of the other supplementation groups. Finally, lipid peroxidation (LPO) levels were significantly elevated by 26% in control *Drosophila* from day 0 to day 30 (Fig. 5d; p < 0.01); which was reduced at day 30 by Lf5221, TFLA, the probiotic and synbiotic formulations. Again, the synbiotic formulation had a significantly higher impact than the probiotic formulation and any of the individual therapies and was the only group which rescued the level of LPO compared to the day 0 controls (p < 0.01). Clearly, the synbiotic formulation has the most robust and consistent impact on markers of oxidative stress indicating its combinatorial action.

**Figure 6:**
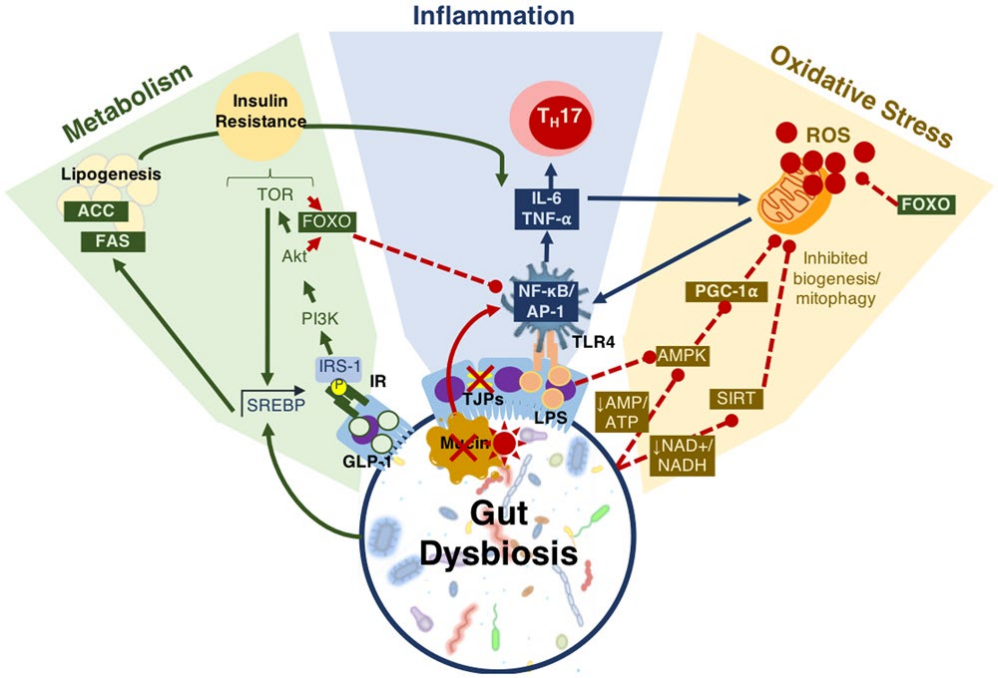
Model of mechanisms of gut microbiota-host communication influencing aging factors. The gut microbiota communicates with the metabolic, inflammatory and oxidative stress pathways via direct and indirect mechanisms. As the physiological changes in all three of these axes are cross-regulatory, the simultaneous action implemented by the gut microbiota makes it a powerful influence in aging and age-related chronic disease development. Abbreviations: glucagon-like peptide (GLP)-1, insulin receptor (IR), insulin receptor substrate (IRS)-1, phosphoinositide 3-kinase (PI3K), protein kinase B (Akt), target of rapamycin (TOR), Forkhead Box O protein (FOXO), sterol regulatory element binding protein (SREBP), acetyl CoA carboxylase (ACC), fatty acid synthase (FAS), tight junction proteins (TJPs), lipopolysaccharide (LPS), toll-like receptor (TLR)4, nuclear factor kappa-light-chain enhancer of activated B cells (NF-kB), activator protein (AP)-1, tumor necrosis factor (TNF)-α, interleukin (IL)-6, T helper (Th)17, AMP-activated protein kinase (AMPK), peroxisome proliferator-activated receptor gamma coactivator (PGC)-1α, sirtuin (SIRT), reactive oxygen species (ROS).

An elevation in oxidative stress is result of increased production of ROS particles due to dysfunctional mitochondria coupled with a reduction in antioxidant capacity. To test the mitochondrial functionality, the activity of each of the ETC complexes were tested before and after supplementation with probiotics and/or prebiotics in aging *Drosophila*. NADH coenzyme Q reductase (ETC complex I) accepts electrons from the Krebs cycle on the electron carrier NADH to transfer them to complex II. ETC complex I’s activity was reduced at days 10, 20 and 30 in control *Drosophila* reaching a 62% decrease in activity by day 30 (Fig. 5e; p < 0.01). Supplementation with TFLA, the probiotic and synbiotic formulations elevated complex I activity at day 30 with the probiotic and synbiotic formulations completely rescuing its activity (p < 0.01). Complex II’s (succinate dehydrogenase) activity was also reduced at days 20 and 30 (Fig. 5f; p < 0.05), though none of the treatments benefited complex II’s activity at day 30. However, the TFLA and synbiotic group reduced ETC complex II’s activity to the level of the day 0 control (p > 0.05. Complex III (cytochrome bc1 complex) activity was also reduced in the control group by 76% at day 30 compared to day 0 (Fig. 5g; p < 0.01). At day 30, every probiotic and/or prebiotic group elicited a beneficial effect on complex III activity; however, the probiotic and synbiotic formulations rescued complex III expression to the level of day 0 controls (p > 0.05). The probiotic formulation had a significantly greater impact than the individual probiotics while the synbiotic formulation was greater than Bi702255. Finally, complex IV (cytochrome c oxidase) activity was similarly reduced by 67% in control *Drosophila* by day 30 (p < 0.01), an effect elevated by all probiotic and/or prebiotic treatments (Fig. 6d; p < 0.05). Treatment with Lf5221, Bi702255 and TFLA rescued complex IV’s activity to the level of day 0 controls however the synbiotic formulation at day 30 had a significantly higher impact than Lp8826 or Lf5221on complex IV’s expression.

## Discussion

Aging and longevity are complex multi-faceted biological processes which are heavily integrated with the composition of the gut microbiota. The power of the gut microbiota comes in its ability to simultaneously influence multiple age-related biological processes including inflammation, oxidative stress, metabolic regulation and energy homeostasis (Fig. 6). It was previously shown that *Drosophila*’s gut microbiota varied significantly with age with a strong expansion of most groups and a disproportionate rise in the pathogenic *Gammaproteobacteria* spp. and *Enterococcus* spp.^28^. This is a known phenomenon in *Drosophila* reflecting age-related immunosenescence and accumulating intestinal barrier dysfunctions from proinflammatory pathobionts^29^. These changes parallel the age-related variations observed in aging humans^14,19^. Treatment with the probiotic and/or prebiotic agents used in the present study were previously shown to reduce the bacterial load in aging *Drosophila*^28^, have immunomodulatory effects^30^, regulate metabolism^31^ and enhance longevity^28^ indicating the potential of developing a probiotic-based therapeutic agent for age-related disease.

There is a significant sexual dimorphism in longevity mechanisms in Drosophila. For example, dietary restriction in Drosophila larvae induced a significant increase in InR gene expression in adult males, but not females^32^ while sexual dimorphisms in mitochondrial maintenance mechanisms including autophagy, Akt, p53 and FOXO in mediating sex-specific differences in stress resistance and aging^33^. Finally, resveratrol was shown to increase longevity in *Drosophila* in a gender- and diet-dependent manner^34^ indicating the importance of distinguishing gender in longevity studies. For this matter, the present study was conducted exclusively in males to reduce the interference of hormonal regulation on typical longevity measures including InR expression, inflammation and oxidative stress.

The synbiotic formulation boosted longevity by 60% compared to *Drosophila* on a conventional diet while the probiotic formulation increased longevity by 55%. There have been a handful of other studies indicating the prolongevity potential of probiotic supplementation^35–38^ though, to the author’s knowledge this is the first study that demonstrates the simultaneous and multi-faceted action of a novel synbiotic formula on several markers of aging attributing to its prolongevity effects.

Suppression of insulin-like growth factor (IGF)-1 and insulin signaling have been identified as the main mechanisms through which calorie restriction leads to an increase in longevity^39^. In the present study, all levels of metabolic distress in aging control *Drosophila* were rescued by the synbiotic formulation including total weight, glucose and triglyceride levels. Previous studies have shown that downregulation of the *dilps* and the insulin receptor increases lifespan in *Drosophila*^40^ and these factors are under dietary regulation^41^.

Regarding the insulin-signaling pathway, elevation of dAkt and dTOR along with the reduction of dFOXO were all rescued by the synbiotic treatment, with beneficial yet variable effects by the individual probiotics and TFLA. Inhibition of TOR signaling with its natural inhibitor rapamycin has been shown to increase longevity in yeast^42^, nematodes^43^, *Drosophila*^44^ and mice^45^ by mimicking the effects of calorie restriction. Downstream of TOR signaling is the FOXO family of transcription factors. AMPK activation by calorie restriction is also linked to elevated FOXO expression and pro-longevity effects^46^ including the simultaneous inhibition of ROS production^47^, NF-κB induction^48^, senescence^49^ and prevention of apoptosis^50^ along with encouragement of the protective mechanisms of mitophagy and autophagy^51^. Several studies have linked polymorphisms of FOXO3 in humans to increased longevity^5,52^ while FOXO3 overexpression in *Drosophila*^53^ and mice^54^ also imparted lifespan extension. Interestingly, several studies indicated that consumption of polyphenols including green tea epigallocatenin, curcumin and resveratrol stimulate FOXO expression and consequently longevity through mechanisms involving increased SOD, GPx and sirtuin 1 expression with decrease in NF-κB, TNF-α, ROS, inflammation and oxidative stress^55,56^. Likewise, in the present study, FOXO expression was upregulated by the synbiotic formulation.

Similar to the glucose-regulating factors, many of the underlying lipogenic factors were also positively affected by the probiotic and synbiotic formulations. There was an improvement of lipogenesis dysregulation, indicated by the rescued expression of *FAS*, *SREBP* and *LSD2* in *Drosophila* supplemented with the synbiotic formulation. The inherent increase in lipogenic *ACC* and *FAS* genes in aging control flies was downregulated most significantly by the probiotic and synbiotic formulations, as was the gluconeogenic PEPCK factor. These changes may be attributed to the transcriptional regulation by SREBP, whose expression was reduced to the level of young flies by both the probiotic and synbiotic formulations. Importantly, SREBP regulation is under the control of the IlS pathway, being activated by both Akt and TOR signaling^57^. The regulation of fatty acid lipogenesis is an important consideration to many aspects of age and age-related conditions, particularly inflammation as obesity is inherently linked with a proinflammatory state^58^ which is known to be preventable with adequate probiotic and prebiotic treatment^59,60^.

It was previously shown that PPARγ mediated responses are central to the synbiotic’s action in metabolic stress models in *Drosophila melanogaster*^31^. PPARγ is highly expressed in the adipose tissue and a key regulator of lipogenesis and adipogenesis^61^ as well as a major insulin sensitizer^62^. Further PPARγ expression in other tissues is thought to regulate their metabolism and the inflammatory response^63^ making it at the hub of many aging hypotheses^64^. In the present study, the *Drosophila* PPARγ target E75 was significantly downregulated in aging control *Drosophila*, an effect that was improved only in the probiotic and synbiotic groups with the latter actually increasing PPARγ expression over time. This effect would explain the beneficial action on the lipogenesis factors and insulin sensitivity supporting the notion that the synbiotic treatment is regulating metabolic stress in aging *Drosophila* at a high level and explaining the broad metabolic effects.

Aging is associated with immunosenescence caused by an exhaustion of stem cells reducing the immune system’s regenerative capacity, accumulation of antigens and thymic atrophy^65^. Dysfunctional immune cells disable the body from mounting an appropriate immune response leading to the accumulation of damaged cells that release proinflammatory cytokines. Chronic low-grade inflammation (inflammaging) is associated with many age-related diseases such as neurodegeneration, cardiovascular disease, insulin resistance, diabetes, osteoporosis, cognitive decline, dementia, frailty, cancer and importantly, mortality (rev. in^66^).

The gut microbiota of elderly persons reflects a pro-inflammatory constitution enriched in *Proteobacteria* spp. and lacking butyrate producing bacteria^2^. With age, the integrity of the GIT epithelial lining becomes compromised allowing the infiltration of bacteria and bacterial products into the host’s bloodstream contributing to inflammaging^67^. All of these factors involve the gut microbiota^68^, and age-related variations in the microbiota reduce gut epithelial integrity and induce intestinal dysplasia^69^. The proinflammatory environment also encourages NF-κB activation through LPS-TLR4 interaction as well as the differentiation of naïve T cells into proinflammatory Th17 cells^70^. The gradual accumulation of chronic low-grade inflammaging could be the source of many age-related chronic diseases as inflammation is comorbid with elevated ROS production, mitochondrial dysfunction and metabolic abnormalities.

As previously shown, there is an upregulation of proinflammatory pathobionts in aging *Drosophila*, which were downregulated by the synbiotic formulation^28^. Indeed, aging *Drosophila* have been shown to be more prone to infection and have an impaired immune system, such as phagocytosis and melanization^71^. A general increase in immune-related genes, increased bacterial loads and more persistent AMP activation after infection resembling the chronic-inflammation state observed in humans has also been observed in *Drosophila*^72^. In the present study, there was an age-related decline in innate immune functionality in control *Drosophila*. In particular, there was a decline in the active immunological agents against both gram-positive (*S. aureus*) and gram-negative (*E. coli*) challenges, an effect that was significantly impacted by all the probiotic treatments, but to the greatest extent, by the probiotic and synbiotic formulations. This could reflect the decreased ability of aging *Drosophila* to mount an immune attack against invading pathogens as previously noted^73^. In contrast to other studies that observed an increase in immune gene expression^71^, a decrease in the expression of *Duox* and *IMD* was observed in aging control flies indicating a weakening of the innate immune response and supporting the weakened immunity to a pathogenic insult as observed in the agar diffusion assay. *Duox* and *IMD* are among the first line of defense of the *Drosophila* innate immune system and regulated directly by antigen recognition of invading pathogens, so, it is possible that the response of the core immune-modulating cells in the fat body is compromised by age affecting the production of *Duox* and *IMD*. This could include the Janus Kinase/Signal Transducer Activator of Transcription (JNK/STAT) pathway which has been shown to have competing or cooperative action on the systemic immune response in *Drosophila*^74^.

Despite the decrease in *IMD* expression in aging *Drosophila*, an increase in AMP expression, particularly *Attacin A*, *Defensin* and *Diptercin*, was observed and dramatically benefitted by both individual probiotic, prebiotic and the probiotic and synbiotic formulation supplementation. This has been previously observed^75^ and directly linked to intestinal barrier dysfunction, which is correlated to lifespan in *Drosophila*^76^. The reason for the discrepancy between the *IMD* signaling and AMP expression could be due to the different levels of regulation of AMP expression. Elevated AMP expression in aging flies is correlated to an increase in oxidative stress^77^. Some of the AMPs including *Attacin A* are co-regulated by inflammatory elements such as the AP-1 and NF-κB proteins as well as HDAC activity^75^. Also, the AMPs are influenced by hormonal signaling, namely 20-hydroxyecdysone and juvenile hormone^78^, which are differentially affected by aging. AMP expression is also impacted by IlS^71^ which could explain the dramatic impact of probiotic treatment on AMP expression in aging *Drosophila*. Indeed, dFOXO was shown to regulate AMP expression, especially in conditions when the Toll and IMD pathways are defective^79^.

Mitochondria progressively lose their energetic capacity with age^80^ along with morphological changes, reduction in numbers, loss of protein quantity and mtDNA mutations^81^. Dysregulation of mitophagy and the mitochondrial fission-fusion cycles also compromises the mitochondrial integrity leading to elevated ROS production and consecutive mitochondrial damage^82^.

A decline in mitochondrial functionality was confirmed in the present study as the activity of each of the ETC complexes in control flies was reduced over time. The mitochondria play a key role in aging and *Drosophila* have been identified as powerful model for studying mitochondrial activity in age^83^. A similar phenomena was observed in various tissues in mice and rats, though decline in activities of only complexes I and IV were observed^84^ while the activity of complexes II and III remained relatively unchanged^85^. TFLA, the probiotic and synbiotic formulations were able to increase ETC complex 1, 3 and 4 activities at day 30 compared to controls, which is very significant in demonstrating how a probiotic treatment can influence mitochondrial complex integrity and consequently the production of ROS particles.

One of the key regulators of mitochondrial biogenesis is the PGC-1 family of transcriptional coactivators, whose expression declines with age^86^. The loss of PGC-1α is associated with a reduction in mitochondrial biogenesis, reduced fission-fusion cycles and dysfunctions in mitophagy^87^. Nutrient deprivation is a key modulator of mitochondrial dynamics as calorie restriction will lower the AMP/ATP and NADH/NAD + ratios which ultimately stimulates mitochondrial biogenesis and activity through PGC-1α and SIRT1, respectively. It was shown in *Drosophila* that overexpression of the PGC-1α homology (dPGC-1/spargel) was sufficient to increase mitochondrial activity and that tissue-specific expression of dPGC-1 in the digestive tract extends longevity^86^. Supporting this, E75, the *Drosophila* PPARγ target, was shown to decline with age in the present study, likely representing the decline in PGC-1α equivalents. The individual probiotic treatment offered little benefit to E75 expression; however, treatment with either the probiotic or synbiotic formulations elevated E75 expression at day 30. This indicates that the management of PPARγ can be one of the critical mechanisms through which the gut microbiota is managing longevity through mitochondrial complex integrity.

ROS production is an essential part of health, though in excess promotes disease. Immunosenescence aggravates redox stress and vice versa stimulating a positive-feedback loop between ROS production and inflammation^88^. ROS levels were significantly elevated in the current aging model as control *Drosophila* saw an increase in total oxidants and LPO levels with significant decreases in SOD and GPx activity. The probiotic and synbiotic formulations had a mild impact on the anti-oxidant enzyme activities, however significantly reduced the levels of total oxidant and LPO, with the synbiotic formulation being more significant in the latter. This is very significant in the context of neurodegeneration as there are a high level of PUFAs in neuronal membranes and the level of LPO in Alzheimer’s disease is correlated with the degree of cognitive impairment^89^.

## Conclusion

With age, the body become less efficient at handling environmental stresses leading to a myriad of physiological imbalances including elevated inflammation, oxidative stress, metabolic dysregulation and mitochondrial damage. There will never be a single therapeutic or dietary solution to manage the mounting chronic diseases associated with aging; however, maintaining a healthy gut microbiota through the use probiotic and prebiotic supplements can delay chronic disease onset and promote longevity by simultaneously affecting each of the main triggers of aging. In the present study, an optimized probiotic formulation containing three bioactive probiotics was combined with a novel polyphenol-rich prebiotic Triphala to create a novel synbiotic formulation that promotes longevity. The synbiotic formulation has a combinatorial effect on various markers of aging including the basic signaling pathways that manage the cross-regulation of these markers. By understanding how the gut microbiota and probiotic treatments intersect with these key aging pathways, specific formulations may be prescribed early in life to prevent chronic disease onset including cardiovascular disease, diabetes, obesity, cancer and even neurodegeneration.

## Methods

### Cultivation of Probiotics

Three probiotic strains, *Lactobacillus plantarum* NCIMB 8826 (Lp8826), *Lactobacillus fermentum* NCIMB 5221 (Lf5221) and *Bifidobacteria longum* spp. *infantis* NCIMB 702255 (Bi702255) were obtained from NCIMB culture collection (Aberdeen, Scotland, UK). Cells were cultured in Man-Rogosa-Sharpe (MRS) media obtained from Sigma Aldrich (Oakville, ON, Canada) at 37 °C on MRS-agar plates or in liquid media. After one round of liquid culture, several bacterial stocks were made in MRS containing 20% (*v/v*) glycerol and stored at −80 °C. As constant culturing was required to carry out all experiments, bacterial stocks were renewed from the frozen stock bi-weekly in order to maintain culture purity. To preform each individual experiment, a 1% (*v/v*) inoculum was used for subculturing, incubated at 37 °C for 18 h and removed immediately before use.

### Probiotic and Synbiotic formulation

The dried components of Triphala (TFLA; *Emblica officinalis, Terminalia bellirica* and *Terminalia chebula*) were obtained from the Ayurvedic Pharmacy at Banaras Hindu University in Varanasi, India. Each component was individually weighed and combined in equal parts (by weight) before being manually crushed and ground with a mortar and pestle. The 5 g of TFLA powder was combined with 1 L of the *Drosophila* media during the boiling process to make a final concentration of 0.5% TFLA in the complete media. The probiotic formulation contained a total of 3.0 × 10^9^ CFU/ml of probiotics with equal distribution between Lp8826 (1.0 × 10^9^ CFU/ml), Lf5221 (1.0 × 10^9^ CFU/ml) and Bi702255 (1.0 × 10^9^ CFU/ml). The synbiotic formulation contained the described probiotic formulation in combination with the 0.5% addition of TFLA powder.

### Drosophila husbandry

Wildtype *Drosophila melanogaster* (Oregon R) were procured from the Bloomington Drosophila Stock Center (Indiana University, Bloomington Indiana). Flies were reared on a standard cornmeal-sucrose-yeast media without active yeast culture prepared by boiling the cornmeal (83 g), sucrose (50 g) and yeast extract (30 g) in distilled water for 30 min. *Drosophila* were kept in controlled conditions with a 12 h:12 h light-dark cycle at 20 °C. In all experiment, male flies were isolated 2–3 days following eclosion to maintain population homogeneity. To prepare the *Drosophila* bottles inoculated with probiotics and/or pathogenic bacteria, the overnight cultures as described were centrifuged at 3000 × *g* for 10 min at 4 °C. The pellet was washed once and resuspended in 0.85% (*w/*v) physiological saline. Total colony counts were determined by spectrophotometry compared to a standard curve prepared with colony forming units (CFUs) on MRS plates. Inoculated media was prepared by partitioning the concentrated bacterial culture into the cooled, yet liquid, media to the indicated final concentrations measured as CFU/ml media. This is a verified method of oral-inoculation to flies as bacterial cells remained viable in the *Drosophila* media for up to two weeks before a detectable loss of concentration by daily CFU counting. Nevertheless, flies were transferred to new inoculated bottles every 3–4 days during the course of an experiment.

### Metabolic measurements in Drosophila

Body weight was assessed by weighing ten male flies in replicates of five at the time of anesthization. Glucose measurements were taken from 5 independent groups from both hemolymph and whole-body homogenates of male *Drosophila* representing the levels of circulating and total glucose, respectively. Hemolymph was extracted by piercing anesthetized *Drosophila* with a fine tungsten needle, placing them in a small tube perforated with several holes situated in a larger tube and centrifuged for 10 min at 6000 × g. For the whole-body homogenates, the total protein content was first determined using a Bradford Assay and resultant quantification of metabolic markers was standardized against the total protein content in order to account for variations in fly mass. Following, the homogenate was heat-treated for 20 min at 70 °C to remove any complexes. Glucose levels were measured in 2 μl of hemolymph or 5 μl of whole-body homogenate using the Glucose (HK) Assay kit (Sigma, Oakvilla, ON, Canada) according to the manufacturer’s instructions. Whole-body triglycerides were determined in 10 μl of whole-body homogenate using the Triglycerides Liquicolor Test Mono (Stanbio, TX, USA) according to the manufacturer’s instructions. At each time point for each treatment group, 5 independent samples of flies were harvested.

### Genetic variation of metabolic markers

RNA was extracted from twenty-five whole flies using Trizol (ThermoFisher, MA, USA) according to the manufacturer’s instructions. cDNA was synthesized from 1 μg of RNA measured with the ND-2000 Nanodrop (FisherScientific, Ottawa, ON, Canada) using the High-Capacity cDNA Synthesis Kit (ThermoFisher, MA, USA) according to the manufacturer’s instructions. Expression of various immunological factors genes was conducted using SybrGreen (EvaGreen qPCR Mastermix, Diamed, Mississauga, Canada) real-time quantitative PCR (Eco Real-Time PCR System, Illumina, CA, USA). Primers, their sequences and annealing temperatures are listed in Supplementary Table 1 and final gene expression was calculated using the 2^ddCT^ method relative to the level of ribosomal protein Rp49. Each sample was an average of 3 technical replicates and statistical measures were conducted with 5 biological replicates.

### Oxidative stress markers

Oxidative stress markers were measured from fresh pooled *Drosophila* homogenates prepared from 5 independent groups of 25 male flies in Tris-EDTA-Triton X-100 buffer, pH 7.4 filtered through a fine cloth to remove solid particles. Total oxidants were assayed using 2′-7′-dichlorofluorescein diacetate (DCFA) (Sigma, Oakville, ON) as previously outlined^90^. Briefly, 20 μl of fly homogenate was mixed with 170 μL of Locke’s buffer. Following, 10 μl of 1 mM DCFA solution was added to each well and after 3 min incubation and fluorescently read at 474 nm excitation and 530 nm emission wavelengths. Quantification was normalized to the amount of protein in each sample.

SOD activity was tested using a xanthine-xanthine oxidase reaction to generate superoxide radicals and nitrotetrazolium blue (NBT) reduction as an indicator of superoxide production^91^. Briefly, the working solution consisted of 110 μl of potassium phosphate buffer with 20 mg/ml BSA, 6.25 μl catalase (40 U/ml), 6.25 μl of NBT and 50 μl of xanthine (1.8 mM). To the working solution, 7.5 μl of homogenate and 20 μl of xanthine oxidase (XOD, 5 U/ml) was added and incubated at 37 °C for 20 min in the dark with agitation to allow colour to develop. Absorbance of reduced NBT was recorded at 570 nm, compared to a standard curve of SOD and normalized to the amount of protein in the sample

GPx activity was assessed based on the oxidation of glutathione (GSSG), which was constantly supplied by an excess of glutathione reductase (GR). To measure GPx activity the consequent reduction of the cosubstrate NADPH was monitored at 340 nm as previously shown^92^ with modifications. Briefly, a GPx buffer was made containing 0.5 M sodium phosphate (pH 7.2), 100 mM EDTA and 1.1 mM sodium azide. The GPx assay buffer consisted of 1.33 mM of GSSH and 1.33 U/ml GR in GPx buffer. The assay solution consisted of 160 μl of GPx assay solution, 10 μl of NADPH (5 mM), 20 μl hydrogen peroxide (0.5%) and 15 μl of homogenate. The absorbance at 340 nm was monitored for 3 min and the linear portion of the curve was assessed for GPx activity and quantification was normalized to the amount of protein in the sample.

The level of lipid peroxidation (LPO) was assessed as bound malondialdehyde (MDA) was hydrolyzed in the presence of butylated hydroxytolene (BHT) as adapted from^93^. The reaction solution contained 10 mM of 1-metyl-2-phenylindole in a 3:1 mixture of acetonitrile:methanol. To 120 μl of this solution, 20 μl of sample and 40 μl of 37% HCl was added and allowed to react at 100 °C for 60 min. The absorbance of the resulting solution was measured at 550 nm, compared to MDA standard solution and normalized to the amount of total protein in the sample.

### Agar disk diffusion assay

To assess the *Drosophila*’s direct ability to inhibit the growth of both *E. coli* and *S. aureus*, hemolymph from 50 male flies from 5 independent groups reared for 10 days on media containing the probiotic and/or prebiotic therapy was placed on a lawn of either *S. aureus* or *E. coli* on Tryptose Sulfite Cycloserine (TSC)-agar (Sigma, Oakville, ON) plates. To isolate hemolymph, 50 flies were pierced with a fine needle in the thorax region and placed in a small Eppendorf tube perforated with several holes, situated in a larger Eppendorf tube. The pierced flies were centrifuged at 3000 × *g*, at 4 °C for 20 min to remove sufficient hemolymph for analysis. The hemolymph (20 μl) was diluted to 50 μl with sterile physiological saline and pipetted onto a 5 mm sterile circle of filter paper situated on the nutrient-agar plates freshly spread with 100 μl of overnight *S. aureus* or *E. coli* solution. The plates inoculated with the hemolymph saturated filter paper were placed at 37 °C for 24 h and the zone of inhibition (diameter) was measured.

### Inflammatory markers

The expression the genetic inflammatory markers was assessed as described in the metabolic markers section, except using the primer sequences outlined in Supplementary Table 2. Each sample was an average of 3 technical replicates and statistical measures were conducted with 5 biological replicates.

### Mitochondrial electron transport chain (ETC) complex activity

Mitochondria was isolated from 5 independent groups of male *Drosophila* by homogenizing 40 anesthetized flies in 4 mL of ice-cold hypotonic buffer (25 mM K_2_HPO_4_ and 5 mM MgCl_2_) with a pre-cooled smooth pestle tissue grinder (Corning, NY, USA) on ice. Large pieces were filtered out using a fine nylon mesh and the remaining isolate was flash-frozen on dry ice before being stored at −80 °C. Before use, the homogenates underwent 3 freeze-thaw cycles to open the mitochondrial membrane.

ETC complex I (NADH – ubiquinone oxidoreductase) activity was assessed by measuring the electron transfer to decyluniquinone from NADH as previously described^78^. The assay buffer contained potassium phosphate buffer (50 mM, pH 7.2) in addition to BSA (50 mg/mL), potassium cyanide (KCN; 10 mM), NADH (10 mM) and rotenone (1 mM). To start the reaction, 25 μL of the homogenate was added to 150 μL of the assay buffer in addition to decyluniquinone (10 μM). The change in absorbance at 340 nm was monitored over two minutes and the slope of NADH reduction was recorded and normalized to the total protein in the sample.

ETC complex II (succinate-ubiquinone oxidoreductase) was measured as the rate of 2,6-dicholorophenolindophenol (DCPIP) reduction as previously described^78^. The assay buffer contained potassium phosphate buffer (50 mM, pH 7.6) in addition to BSA (50 mg/mL), potassium cyanide (KCN, 10 mM), succinate (400 mM) and DCPIP (0.015% *w*/*v*). To start the reaction, 10 μL of the homogenate in addition to phenazine methosulfate (65 mM) was added to 160 μL of the assay buffer. The rate of reduction was measured at 600 nm over two minutes and the activity of ETC complex II was measured as the linear portion of the slope of DCPIP reduction and normalized to the amount of protein in the sample.

ETC complex III (NADH-cytochrome c oxidoreductase) activity was determined as the rate of antimycin dependent reduction of cytochrome c as previously described^78^. The assay buffer contained potassium phosphate buffer (50 mM, pH 7.2) in addition to oxidized cytochrome c (1 mM), KCN (10 mM), EDTA (5 mM), Tween-20 (2.5%) and antimycin A (1 mg/mL). To start the reaction, 20 μL of sample homogenate was added to the assay buffer in addition to 10 μL of decyubiquiol (10 mM). The reduction of cytochrome c was monitored at 550 nm for 2 min both with and without antimycin A and the difference in the reaction rates was taken as the antimycin A sensitive complex activity and normalized to the amount of protein in the sample.

Finally, ETC complex IV (cytochrome c oxidase) activity was determined by monitoring the rate of cytochrome c oxidation. The assay buffer contained potassium phosphate buffer (50 mM, pH 8.0) supplemented with reduced cytochrome c (1 mM). Cytochrome c was reduced by adding a few grains of sodium dithionite to the oxidized cytochrome c solution until the red colour turned to orange and an absorbance ratio greater of 550 nm to 565 nm was greater than 6. The reaction was initialized with the addition of 25 μl of the homogenate and the rate of reduction recorded for 3 min at 550 nm. The complex rate was normalized to the total amount of protein in the sample.

### Statistics

All statistical analyses were conducted using [R] software. Variations in body weight and motility over the dose-curves of probiotic were assessed with a one-way ANOVA analyses with Tukey post-hoc analyses. Significance of metabolic markers, genetic expression, inflammatory markers, oxidative stress markers and mitochondrial complexes were assessed with a two-way ANOVA in the form Y = µ + *T* + *D* + *T* x *D* + ε where *T* and *D* are treatment and time in days, respectively and ε is the residual variance. Multiple comparisons were conducted between groups using Tukey’s post hoc analyses and significance between the time points within a treatment group is marked as *p < 0.05 and **p < 0.01 whereas significant differences with a timepoint between treatment groups is indicated with τ p < 0.05. A summary of all the main effects is presented in Supplementary Table 3.

### Data availability

All relevant data are contained within the manuscript. Additional raw data will be available upon request.

## Electronic supplementary material

Supplementary Information
