# Supplementary material for: Longevity extension in Drosophila through gut-brain communication
Source: Sci Rep. 2018 May 30;8:8362. doi: 10.1038/s41598-018-25382-z (PMC5976768; doi:10.1038/s41598-018-25382-z)
Supplement: Supplementary file 1 — Supplementary Information [file 41598_2018_25382_MOESM1_ESM.docx]

**Longevity extension in Drosophila through gut-brain communication**

Susan Westfall, Nikita Lomis, Satya Prakash*

Biomedical and Cell Therapy Research Laboratory,

Department of Biomedical Engineering,

McGill University, 3775 University Street, Room 322, Montreal, QC, H3A2B4, Canada.

susan.westfall@mail.mcgill.ca; nikita.lomis@mail.mcgill.ca;

* Corresponding Author: satya.prakash@mcgill.ca; Tel.: 1-514-398-3676. Fax: 1-514-398-7461

**Supplementary Information**

**Supplementary Table 1: Primer sequences of *Drosophila melanogaster* metabolic markers**

| **Gene Name** | **Sequence** | **Annealing Temp.** | **Reference** |
| --- | --- | --- | --- |
| *Dilp 2* | *F: 3’ –* AGCAAGCCTTTGTCCTTCATCTC – 5’ | 50 °C | ^1^ |
|  | *R: 3’ –* ACACCATACTCAGCACCTCGTTG – 5’ |  |  |
| *Dilp 3* | *F: 3’ –* TGTGTGTATGGCTTCAACGCAATG – 5’ | 50 °C | ^1^ |
|  | *R: 3’ –* CACTCAACAGTCTTTCCAGCAGGG – 5’ |  |  |
| *InR* | *F: 5’ –* AACAGTGGCGGATTCGGTT – 3’ | 54 °C | ^2^ |
|  | *R: 5’ –* TACTCGGAGCATTGGAGGCAT – 3’ |  |  |
| *ACC* | *F: 3’ –* TTAGTCAGCTGCAGGCAAAGG – 5’ | 54 °C | ^3^ |
|  | *R: 3’ –* CGGAAGCTAACGCCACACA – 5’ |  |  |
| *FAS* | *F: 3’ –* CAACAAGCCGAACCCAGATCTT – 5’ | 50 °C | ^3^ |
|  | *R: 3’ –* CAAAGGAGTTCAGGCCGATGAT – 5’ |  |  |
| *PEPCK* | *F: 3’ –* CGCCCAGCGACATGGATGCT – 5’ | 60 °C | ^4^ |
|  | *R: 3’ –* GTACATGGTGCGACCCTTCA – 5’ |  |  |
| *dTOR* | *F: 3’ –* GGCCGTCCAGGTTCAAAAAC - 5’ | 59 °C | *This study* |
|  | *R: 3’ –* AATCCGGCGATAGTTCCGTC – 5’ |  |  |
| *dAkt* | *F: 3’ –* GAGTCGTGTGCTCAAGTCCA – 5’ | 59 °C | *This study* |
|  | *R: 3’ –* TGCATCACAAAACACAGGCG – 5’ |  |  |
| *dFOXO* | *F: 3’ –* TCGCCGAACTCAGTAACCAC – 5’ | 59 °C | *This study* |
|  | *R: 3’ –* TCCTATCAAAGTAGAGGCGCA – 5’ |  |  |
| *SREBP* | F: 5’-GGCAGTTTGTCGCCTGATG-3’ | 56 °C | ^5^ |
|  | R: 5’-CAGACTCCTGTCCAAGAGCTGTT-3’ |  |  |
| *E78* | F: 5’-CAGTGTCTCTCGTTGCTCA-3’ | 54 °C | *This study* |
|  | R: 5’-AACCGATTGCTTCGCTCTCT-3’ |  |  |
| *LSD* | F: 5’-ACTTGTAGTGCCAGTTCCCG-3’ | 52 °C | *This study* |
|  | R: 5’-ACCAGACTGCTCCACATTCG-3’ |  |  |
| *Rp49* | *F: 3’ –* AGATCGTGAAGAAGCGCACCAAG – 5 ‘ | 52 °C | ^6^ |

**Supplementary Table 2: Primer sequences of *Drosophila melanogaster* inflammatory markers**

| **Gene Name** | **Sequence** | **Annealing Temp** | **Reference** |
| --- | --- | --- | --- |
| *Duox* | *F:* GCTGCACGCCAACCACAAGAGACT | 54 °C | ^5^ |
|  | *R:* CACGCGCAGCAGGATGTAAGGTTT |  |  |
| *IMD* | *F:* TCGAATGCCAATAATCTGCA | 52 °C | ^5^ |
|  | *R:* CGCGATGCTGGGACTCCCAC |  |  |
| *Relish* | *F:* TGGGAGGCATACGCAAAGT | 55 °C | ***This study*** |
|  | *R:* CAATTACGCTCCGTGGCTTG |  |  |
| *Attacin A* | *F:* GGCCCATGCCAATTTATTCA | 56 °C | ^7^ |
|  | *R:* CATTGCGCTGGAACTCGAA |  |  |
| *Diptericin* | *F:* AGGTGTGGACCAGCGACAA | 56 °C | ^7^ |
|  | *R:* TGCTGTCCATATCCTCCATTCA |  |  |
| *Defensin* | *F:* GCACAATGAAGTTCACCATCGT | 56 °C | ^7^ |
|  | *R:* CCACACCCATGGCAAAAAC |  |  |

**Supplementary Table 3: Summary of Statistical Main Effects**

| **Experiment** | **Source** | **df** | **F** | **p** |
| --- | --- | --- | --- | --- |
| Longevity (Fig. 1a) | Treatment | 70 | 1844 | P < 0.0001 |
|  | Time | 4 | 397 | P < 0.0001 |
|  | Treatment X Time | 280 | 31.88 | P < 0.0001 |
| Weight (Fig. 1b) | Treatment | 6 | 80.09 | P < 0.001 |
|  | Time | 3 | 1774 | P < 0.001 |
|  | Treatment X Time | 18 | 20.98 | P < 0.001 |
| Glucose (Fig. 1c) | Treatment | 6 | 57.97 | P < 0.0001 |
|  | Time | 3 | 103 | P < 0.0001 |
|  | Treatment X Time | 18 | 18.96 | P < 0.0001 |
| Triglyceride (Fig. 1d) | Treatment | 6 | 68.23 | P < 0.0001 |
|  | Time | 3 | 117 | P < 0.0001 |
|  | Treatment X Time | 18 | 5.34 | P < 0.0001 |
| *Dilp 2* (Fig. 2a) | Treatment | 6 | 3.621 | P < 0.05 |
|  | Time | 3 | 81.31 | P < 0.0001 |
|  | Treatment X Time | 18 | 3.47 | P < 0.0001 |
| *Dilp 3* (Fig. 2b) | Treatment | 6 | 1.35 | P > 0.05 |
|  | Time | 3 | 7.11 | P < 0.01 |
|  | Treatment X Time | 18 | 4.87 | P < 0.0001 |
| InR (Fig. 2c) | Treatment | 6 | 8.014 | P < 0.0001 |
|  | Time | 3 | 207.7 | P < 0.0001 |
|  | Treatment X Time | 18 | 4.53 | P < 0.0001 |
| dAkt (Fig. 2d) | Treatment | 6 | 9.12 | P < 0.0001 |
|  | Time | 3 | 87.23 | P < 0.0001 |
|  | Treatment X Time | 18 | 4.82 | P < 0.0001 |
| dTOR (Fig. 2e) | Treatment | 6 | 5.43 | P < 0.0001 |
|  | Time | 3 | 62.35 | P < 0.0001 |
|  | Treatment X Time | 18 | 4.91 | P < 0.0001 |
| dFOXO (Fig. 2f) | Treatment | 6 | 30.22 | P < 0.0001 |
|  | Time | 3 | 14.81 | P < 0.001 |
|  | Treatment X Time | 18 | 4.12 | P < 0.0001 |
| ACC (Fig. 3a) | Treatment | 6 | 6.28 | P < 0.0001 |
|  | Time | 3 | 10.12 | P < 0.0001 |
|  | Treatment X Time | 18 | 4.12 | P < 0.0001 |
| FAS (Fig. 3b) | Treatment | 6 | 4.92 | P < 0.0001 |
|  | Time | 3 | 9.34 | P < 0.0001 |
|  | Treatment X Time | 18 | 3.91 | P < 0.0001 |
| SREBP (Fig. 3c) | Treatment | 6 | 19.23 | P < 0.0001 |
|  | Time | 3 | 9.12 | P < 0.0001 |
|  | Treatment X Time | 18 | 3.2 | P < 0.0001 |
| PEPCK (Fig. 3d) | Treatment | 6 | 29.34 | P < 0.0001 |
|  | Time | 3 | 4.12 | P < 0.0001 |
|  | Treatment X Time | 18 | 4.63 | P < 0.0001 |
| LSD2 (Fig. 3e) | Treatment | 6 | 27.34 | P < 0.0001 |
|  | Time | 3 | 5.99 | P < 0.0001 |
|  | Treatment X Time | 18 | 3.28 | P < 0.0001 |
| E75 (Fig. 3f) | Treatment | 6 | 18.23 | P < 0.0001 |
|  | Time | 3 | 8.24 | P < 0.0001 |
|  | Treatment X Time | 18 | 5.09 | P < 0.0001 |
| E. coli (Fig. 4a) | Treatment | 6 | 67.43 | P < 0.0001 |
|  | Time | 3 | 605.2 | P < 0.0001 |
|  | Treatment X Time | 18 | 13.98 | P < 0.0001 |
| S. aureus (Fig. 4b) | Treatment | 6 | 54.23 | P < 0.0001 |
|  | Time | 3 | 119.2 | P < 0.0001 |
|  | Treatment X Time | 18 | 14.12 | P < 0.0001 |
| *Duox* (Fig. 4c) | Treatment | 6 | 15.43 | P < 0.0001 |
|  | Time | 3 | 6.04 | P < 0.0001 |
|  | Treatment X Time | 18 | 3.78 | P < 0.0001 |
| IMD (Fig. 4d) | Treatment | 6 | 9.87 | P < 0.0001 |
|  | Time | 3 | 6.15 | P < 0.0001 |
|  | Treatment X Time | 18 | 4.71 | P < 0.0001 |
| Relish (Fig. 4e) | Treatment | 6 | 3.65 | P > 0.05 |
|  | Time | 3 | 6.91 | P < 0.05 |
|  | Treatment X Time | 18 | 2.54 | P > 0.05 |
| Attacin A (Fig. 4f) | Treatment | 6 | 14.32 | P < 0.0001 |
|  | Time | 3 | 8.76 | P < 0.0001 |
|  | Treatment X Time | 18 | 3.73 | P < 0.0001 |
| Defensin (Fig. 4g) | Treatment | 6 | 11.23 | P < 0.0001 |
|  | Time | 3 | 9.51 | P < 0.0001 |
|  | Treatment X Time | 18 | 4.13 | P < 0.0001 |
| Diptercin (Fig. 4h) | Treatment | 6 | 6.987 | P < 0.01 |
|  | Time | 3 | 56.3 | P < 0.0001 |
|  | Treatment X Time | 18 | 2.31 | P > 0.05 |
| Total oxidants (Fig. 5a) | Treatment | 6 | 10.57 | P < 0.0001 |
|  | Time | 3 | 195.6 | P < 0.0001 |
|  | Treatment X Time | 18 | 3.78 | P < 0.0001 |
| SOD activity (Fig. 5b) | Treatment | 6 | 12.34 | P < 0.0001 |
|  | Time | 3 | 87.61 | P < 0.0001 |
|  | Treatment X Time | 18 | 3.92 | P < 0.0001 |
| GPx activity (Fig. 5c) | Treatment | 6 | 11.64 | P < 0.0001 |
|  | Time | 3 | 47.31 | P < 0.0001 |
|  | Treatment X Time | 18 | 4.08 | P < 0.0001 |
| LPO levels (Fig. 5d) | Treatment | 6 | 16.43 | P < 0.0001 |
|  | Time | 3 | 109.23 | P < 0.0001 |
|  | Treatment X Time | 18 | 5.31 | P < 0.0001 |
| ETC Complex 1 (Fig. 6a) | Treatment | 6 | 54.23 | P < 0.0001 |
|  | Time | 3 | 9.12 | P < 0.0001 |
|  | Treatment X Time | 18 | 4.31 | P < 0.0001 |
| ETC Complex 2 (Fig. 6b) | Treatment | 6 | 34.23 | P < 0.01 |
|  | Time | 3 | 7.12 | P < 0.05 |
|  | Treatment X Time | 18 | 2.53 | P > 0.05 |
| ETC Complex 3 (Fig. 6c) | Treatment | 6 | 31.65 | P < 0.0001 |
|  | Time | 3 | 14.32 | P < 0.0001 |
|  | Treatment X Time | 18 | 3.98 | P < 0.01 |
| ETC Complex 4 (Fig. 6d) | Treatment | 6 | 34.21 | P < 0.001 |
|  | Time | 3 | 95.12 | P < 0.001 |
|  | Treatment X Time | 18 | 4.01 | P < 0.001 |

**References Supplementary Information**

1. Luo, J., Lushchak, O. V., Goergen, P., Williams, M. J. & Nassel, D. R. Drosophila insulin-producing cells are differentially modulated by serotonin and octopamine receptors and affect social behavior. **9,** e99732 (2014).

2. Storelli, G. *et al.* Lactobacillus plantarum Promotes Drosophila Systemic Growth by Modulating Hormonal Signals through TOR-Dependent Nutrient Sensing. *Cell Metabolism* **14,** 403–414 (2011).

3. Okamura, T., Shimizu, H., Nagao, T., Ueda, R. & Ishii, S. ATF-2 regulates fat metabolism in Drosophila. *Mol Biol Cell* **18,** 1519–1529 (2007).

4. Porstmann, T. *et al.* SREBP activity is regulated by mTORC1 and contributes to Akt-dependent cell growth. *Cell Metabolism* **8,** 224–236 (2008).

5. Ha, E.-M. *et al.* Coordination of multiple dual oxidase–regulatory pathways in responses to commensal and infectious microbes in drosophila gut. *Nature Publishing Group* **10,** 949–957 (2009).

6. Westfall, Lomis, N., Singh, S. P. & Prakash, S. Ferulic acid produced by Lactobacillus fermentum NCIMB 5221 reduced symptoms of metabolic syndrome in Drosophila melanogaster. *Microbial Biochem Technol* **8,** 272–284 (2016).

7. Tsai, C. W., McGraw, E. A., Ammar, E.-D., Dietzgen, R. G. & Hogenhout, S. A. Drosophila melanogaster mounts a unique immune response to the Rhabdovirus sigma virus. *Applied and Environmental Microbiology* **74,** 3251–3256 (2008).
